# Acetone leaf extracts of some South African trees with high activity against *Escherichia coli* also have good antimycobacterial activity and selectivity index

**DOI:** 10.1186/s12906-017-1831-z

**Published:** 2017-06-19

**Authors:** Ishaku L. Elisha, Francien S. Botha, Balungile Madikizela, Lyndy J. McGaw, Jacobus N. Eloff

**Affiliations:** 1 Phytomedicine Programme, Department of Paraclinical Sciences, Faculty of Veterinary Science, University of Pretoria, Private Bag X04, Onderstepoort, Pretoria, 0110 South Africa; 2 Permanent address: Drug Development Section, Biochemistry Division, National Veterinary Research Institute, P.M.B. 01 Vom, Plateau State, Vom, Nigeria

**Keywords:** Antimycobacterial, Plant extracts, Minimum inhibitory concentration, Selectivity index

## Abstract

**Background:**

Tuberculosis is a world-wide problem affecting humans and animals. There is increasing development of resistance of the pathogens to current antimycobacterial agents. Many authors have investigated activities of extracts and isolated compounds from plants. The traditional uses of plants have frequently been the criterion to select plants investigated. In this contribution, we investigate whether plant extracts with very good activity against *Escherichia coli* may also be active against mycobacteria.

**Methods:**

The antimycobacterial activity of eight South African tree leaf extracts with high activity against *Escherichia coli* were determined in vitro against *Mycobacterium smegmatis, M. fortuitum and M. aurum*, using a serial microdilution method. The cellular cytotoxicity was also determined by the MTT assay using Vero monkey kidney cells. The selectivity index was determined by dividing the cytotoxicity of extracts by MIC.

**Results:**

The antimycobacterial activity of the extracts ranged from 0.02 to 2.5 mg/ml. *Mycobacterium smegmatis* was more sensitive to the extracts (Average MIC = 0.96 mg/ml) and *Mycobacterium aurum* was comparatively resistant (Average MIC = 2.04 mg/ml). The extracts of *Cremaspora triflora* had strong antimycobacterial activity with a MIC of 0.05 mg/ml that compared reasonably well with that of streptomycin (0.01 mg/ml) and rifampicin (0.03 mg/ml), *p* > 0.05. *Cremaspora triflora* had the best selectivity index of 2.87 against *Mycobacterium fortuitum.*

**Conclusion:**

The high activity of *C. triflora* extracts against the fast-growing mycobacteria and good cellular safety is promising. It may be interesting to investigate extracts against pathogenic *M. tuberculosis, M. bovis* and *M. avium* cultures and to isolate active antimycobacterial compounds.

## Background

Tuberculosis (TB) is a highly infectious disease, caused by *Mycobacterium tuberculosis*. TB typically affects the lungs but can also affect other organs in the body [1]. An estimated 5–15% of the 2–3 billion people exposed to *M. tuberculosis* will develop the disease in their lifetime. The probability is even higher among people infected with HIV [1, 2]. In 2014, about 9.6 million people were infected with TB and 1.5 million consequently died. A high proportion of the reported cases (28%) came from Africa [1]. The growing incidence of pathogenic mycobacterial multi-drug resistance to the best two first line antituberculosis drugs - streptomycin and isoniazid and extensively-drug resistance to both first and second line antituberculosis drugs that include fluoroquinolones and capreomycin highlights the critical need to search for newer anti-tuberculosis drugs. There are reports of the emergence of a ‘totally drug-resistant TB’ strain with a limited chance of successful treatment [1].

Natural products, either as a source of pure compounds or as standardised plant extracts, provide opportunities for new drug leads due to the high chemical diversity in plants [3]. It has been shown that extracts of *Maerua edulis*, *Securidaca longipedunculata*, *Zanthoxylum capense* and *Tabernaemontana elegans* have high activity against *Mycobacteria spp*. [4, 5].

To select plants for investigation, the traditional use can be considered. Because traditional healers have mainly water available and antimicrobial compounds are not readily soluble in water [6] this is not such a promising approach [7]. Random screening of tree leaf extracts indicated that many plant extracts have excellent activities [8]. The cell wall of *Mycobacterium tuberculosis* has similar characteristics to both Gram-positive and Gram-negative bacteria [9]. In addition, *M. tuberculosis* is closer related to Gram-negative bacteria than Gram-positive bacteria. Analysis of evolutionary distance between nearest ancestral units, suggest that *M. tuberculosis* is closely related to *Escherichia coli* and *Pseudomonas aeruginosa* [9]. Thus in this study we selected eight plant species with high antimicrobial activity against *Escherichia coli* to determine the potency, efficacy and safety of acetone tree leaf extracts against three fast growing *Mycobacterium* species. With one exception no previous studies were found on the antimycobacterial activity of extracts of these species.

## Methods

### Collection of plant material, drying and storage

Leaves of the selected tree species were collected and dried in the shade before grinding to a fine powder. The trees selected with family and voucher specimen numbers in brackets were *Hypericum roeperianum* G.W. Schimp.ex A. Rich. var. *roeperianum,* (Hypericaceae, PRU 120126)*, Cremaspora triflora* (Thonn.) K. Schum (Rubiaceae, PRU 120129)*, Heteromorpha arborescens* (Spreng.) Chan. & Schltdl (Apiaceae, PRU 120026)*, Bolusanthus speciosus* (H. Bolus) Harms (Fabaceae, PRU 120027)*, Calpurnia aurea* (Aiton) Benth ssp. *aurea* (Fabaceae, PRU 120125)*, Maesa lanceolata* Forssk (Maesaceae, PRU120125)*, Elaeodendron croceum* (Thunb.) DC (Celastraceae, PRU 120127) and *Morus mesozygia* Stapf ex A. Chev (Moraceae, PRU 120128) were collected in 2013, at the University of Pretoria, Botanical Garden, Pretoria National Botanical Garden and Lowveld National Botanical Garden in South Africa. The identity of the trees was confirmed from the tree labels. Voucher specimens were prepared and deposited in collaboration with the herbarium curator Mrs. Elsa van Wyk in the HGWJ Schweickerdt Herbarium of the University of Pretoria (PRU).

### Preparation of extracts

Ground dry leaf powder (1.0 g) of each plant was poured into 50 ml polyester centrifuge tubes and extracted with 30 ml acetone for one hour [10]**.** After this they were centrifuged at 4000 x g for 10 min. The supernatants were decanted into preweighed glass vials through Whatman No. 1 filter paper and concentrated to dryness under a stream of cold air. The dried extracts were made up to a concentration of 10 mg/ml (stock solution) in acetone to be used in subsequent assays and stored at 5 °C in tightly stoppered glass tubes.

### Antimycobacterial activity assay

#### Mycobacterial cultures

*Mycobacterium smegmatis* (ATCC 1441), *Mycobacterium aurum* (NCTC 10437) and *Mycobacterium fortuitum* (ATCC 6841) were cultured as described by McGaw et al. [3], and maintained on Löwenstein–Jensen agar slants, supplemented with glycerol. Inocula suspensions were prepared by mixing a few microbial colonies with sterile distilled water to render a concentration of cells equal to standard 1 McFarland solution (approximately 4 × 10^7^ cfu/ml). The suspension was then diluted with freshly prepared Middlebrook 7H9 broth supplemented with 10% oleic acid, albumin, dextrose, and catalase (OADC) to obtain a final inoculum density of approximately 4 × 10^5^ cfu/ml. A serial microplate broth microdilution technique [11] was used to obtain the MIC values of the various extracts.

#### Cytotoxic activity

The 3-(4,5-dimethylthiazol-2-yl)-2, 5-diphenyltetrazolium bromide (MTT) reduction assay [12] was used to determine the cytotoxicity of the extracts against Vero cells.

### Statistical analysis

All experiments were conducted in triplicate and values expressed as the mean ± standard deviation. Variations in mean were analysed using one-way analysis of variance (ANOVA), and means were statistically significant if *p* < 0.05.

## Results and discussion

There are several reasons given to justify the constant search for new anti-TB drugs to improve the current treatment regimen by reducing therapy time and addressing drug resistance especially against multi-drug resistant (MDR) and extreme drug resistant (XDR) mycobacterial strains. Development of natural anti-TB drugs with minimal hepatotoxic and nephrotoxic effects could add to the collection of available drugs used in TB treatment. In many other human diseases very useful drugs have been discovered from plants. Not any of the drugs (isoniazid, rifampicin, capreomycin, fluoroquinolones, kanamycin and amikacin) used as first or second line drugs in the chemotherapy of TB has its origin from plant derived natural products [13].

The choice of using fast growing and non-pathogenic *Mycobacterium spp.* in antimycobacterial assays was based on their avirulent nature and similarity in sensitivity to pathogenic *Mycobacterium* strains [14, 15]. In addition, there are published reports on the use of *M. fortuitum* as an alternative screening model to *Mycobacterium tuberculosis* for potential antitubercular drug development [14]. Subsequently, Aro et al. [16] found that *M. aurum* is the best predictor of the activity against pathogenic *M. tuberculosis* with a correlation coefficient of 0.9 In addition, *Mycobacterium smegmatis* was the best predictor strain to substitute pathogenic *M. bovis* and *M. tuberculosis*, MIC values obtained using *M. fortuitum* correlated well with those of *M. bovis* BCG [3].

In addition to the antimycobacterial activity, the total activity was also calculated by dividing the mass in mg extracted from 1 g of dried material with the MIC in mg/ml (7). The total activity indicates the volume to which the extract from one g of plant material can be diluted and still inhibit the growth of the microorganism.

The MIC of activity of the extracts against *M. smegmatis* ranged from 0.04 to 2.5 mg/ml. *C. triflora* extracts had the best activity (MIC 0.04 mg/ml), while *M. lanceolata* had moderate activity (MIC 0.16 mg/ml); *H. roeperianum* and *M. mesozygia* had weak activity with MIC = 0.63 mg/ml. The other three species had very low activity. *C. triflora* had the second best total antimycobacterial activity (TAA) value of 504 ml/g and an SI value of 1.44. TAA value of 504 ml/g infers that if 1 g of the dry acetone crude extract is diluted with 504 ml of water it will still inhibit the growth of the targeted microorganism [10]. Other extracts had relatively poor activity against *M. smegmatis* and SI less than 1.0 (Table 1).

**Table 1:** Minimum inhibitory concentration (MIC) and total antimycobacterial activity (TAA) and selectivity index (SI) of the eight selected acetone leaf extracts against *Mycobacterium smegmatis*, *Mycobacterium fortuitum*, *Mycobacterium aurum*. The SI and TAA values of the extracts were calculated from the cytotoxicity and percentage yield of the extracts results published in Elisha et al. [21]

	*M. smegmatis*			*M. fortuitum*			*M. aurum*		
Plants	MIC (mg/ml)	TAA (ml/g)	SI	MIC (mg/ml)	TAA (ml/g)	SI	MIC (mg/ml)	TAA (ml/g)	SI
*Hypericum roeperianum*	0.63	190.42	0.11	1.25	95.97	0.05	1.25	95.97	0.05
*Cremaspora triflora*	0.04	504.17	1.44	0.02	1008.33	2.87	0.08	252.08	0.72
*Heteromorpha arborescens*	1.25	20.83	0.06	2.5	10.41	0.03	2.5	10.41	0.03
*Bolusanthus speciosus*	1.25	18.43	0.04	1.25	18.43	0.04	2.5	9.21	0.02
*Calpurnia aurea*	1.25	22.91	0.01	1.25	22.91	0.01	2.5	11.45	0.01
*Maesa lanceolata*	0.16	695.21	0.01	0.31	358.82	0.01	2.5	44.49	0
*Elaeodendron croceum*	2.5	35.99	0	1.25	71.97	0	2.5	35.99	0
*Morus mesozygia*	0.63	29.31	0.06	0.31	59.57	0.13	2.5	7.39	0.02
Mean	0.96	NA	NA	1.02	NA	NA	2.04	NA	NA
SD	0.74	NA	NA	0.74	NA	NA	0.85	NA	NA
Streptomycin	0.02	NA	NA	0.01	NA	NA	0.01	NA	NA
Rifampicin	0.06	NA	NA	0.02	NA	NA	0.01	NA	NA

*NA* Not applicable

The MIC values of the extracts against *M. fortuitum* ranged from 0.02 mg/ml to 2.5 mg/ml. *Cremaspora triflora* again had the best activity (MIC = 0.02 mg/ml), while *M. lanceolata* and *M. mesozygia* had moderate activity with MICs of 0.31 mg/ml. *C. triflora* extracts had the best total antimycobacterial activity against *M. fortuitum* with a value of 1008 ml/g, and a good selectivity index value of 2.87 (Table 1).

*M. aurum* was much more resistant to the plant extracts and only *C. triflora* had good activity with an MIC value of 0.08 mg/ml, and total antimycobacterial activity value of 255 ml/g and a low SI value of 0.72 (Table 1).

These results were more or less in line with the results of McGaw et al. [3] who investigated the activity of compounds isolated from *Euclea* species against different *Mycobacterium* species. It is possible that the fast growing *Mycobacterium* species are more susceptible than the slow growing species. It also appeared that *M. smegmatis* would be the best predictor of activity against pathogenic mycobacteria [3].

Palgrave (1997) in Erastus et al. [17] reported the traditional use of the dried bark of *Bolusanthus speciosus* in the treatment of tuberculosis. Our results investigating the leaves did not support this claim. The acetone leaf extracts of *Bolusanthus speciosus* had low antimycobacterial activity with a mean MIC value of 1.67 mg/ml against the three tested microorganisms. It is not strange that in vitro studies do not confirm traditional use of plant extracts. This could be due to several reasons. Different extractants could be used, microbial infections could influence activity of extracts left without refrigeration for some time. Clinical identification of the disease and recovery as well as the placebo effect could also have an influence. The activity of plant extracts may also change during different seasons and there could be synergistic activities if extracts of more than one plant species is used.

*Maesa lanceolata,* traditionally used to treat sore throat and influenza [18, 19], had moderate activity against *M. smegmatis* and *M. fortuitum* with MIC values of 0.16 mg/ml and 0.31 mg/ml respectively (Table 1). This may support its use traditionally in the treatment of respiratory infections.

**Fig. 1:**
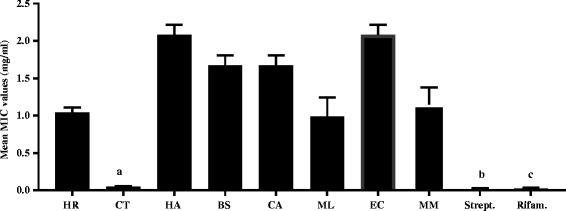
The mean MIC values (mg/ml) of acetone leaf extracts of the different medicinal plants and the positive controls (Streptomycin and Rifampicin) against *M. smegmatis*, *M. fortuitum* and *M. aurum.* Legend: ^a, b and c^ = no statistical significant difference in the mean MIC values, *p* < 0.05. HR = *Hypericum roeperianum*, CT = *Cremaspora triflora*, HA = *Heteromorpha arborescens*, BS = *Bolusanthus speciosus,* CA = *Calpurnia aurea*, ML = *Maesa lanceolata*, EC = *Elaeodendron croceum*, MM = *Morus mesozygia,* Strept. =Streptomycin, Rifam. = Rifampicin

**Fig. 2:**
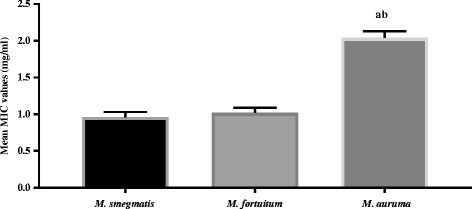
Sensitivity of the tested *Mycobacterium spp.* to the different plant acetone leaf extracts. Legend: ^ab^ = statistically significant difference in mean MIC, *p* < 0.05, when the sensitivity of *M. aurum* was compared to the MIC values of *M. smegmatis* and *M. fortuitum*

*Cremaspora triflora* had the best activity against all the *Mycobacterium spp.* with a mean MIC value of 0.05 ± 0.01 mg/ml and *E. croceum* had the weakest activity against the microbes with MIC value of 2.5 ± 0.41 mg/ml (Fig. 1). *Mycobacterium smegmatis* was more sensitive to the extracts than *M. fortuitum* and *M. aurum* (Fig. 2), however the difference in the mean between *M. smegmatis* and *M. fortuitum* was not statistically significant *p* > 0.05 (Fig. 2). Means of *M. smegmatis* and *M. fortuitum* differed significantly from that of *M. aurum*, *p* < 0.05. Our findings agree with a previous report [20] that *M. smegmatis* was more sensitive to the essential oil of the gall of *Pistacia atlantica* followed by *M. fortuitum,* and *M. aurum.*

The extracts of *C. triflora* have potential as a good candidate for elaborate antimycobacterial investigation. Results from this study on the good activity of the extracts of *C. triflora* (Table 1) against the different mycobacterial strains differs with the report that the extracts of *C. triflora* had moderate activity against *M. smegmatis* and *M. aurum*, with MIC values of 0.23 mg/ml and 0.10 mg/ml respectively [16]. The discrepancies on the potency report of *C. triflora* extracts in our study and that of Aro et al. [16], may be attributed to differences in the season and time of plant collection and storage conditions. It is however agreed that the plant has potential for development as an alternative anti-TB drug.

## Conclusions

Approximately 88% of all the test acetone leaf extracts had weak activities against the non-pathogenic mycobacterial species. This indicates that high activity against *Escherichia coli* is not well correlated with antimycobacterial activity. *Cremaspora triflora* extracts were exceptional, with significant activity against the three fast-growing mycobacterial organisms. *Mycobacterium aurum* was more resistant to the different extracts. The excellent antibacterial activities and selectivity index of the extracts of *C. triflora* makes it the preferred species for in depth pharmacological and biological investigations.

## Abbreviations

HIV: Human Immunodeficiency Virus
MDR: Multidrug resistant
MIC: Minimum inhibitory concentration
MTT 3-(4,5-dimethylthiazol-2-yl)-2: 5-diphenyltetrazolium bromide
TAA: Total antimycobacterial activity
TB: Tuberculosis
XDR: Extreme drug resistant
